# Effect of calcitonin gene-related peptide antagonist on the mortality by nitrate induced tolerance rats with acute myocardial infarction

**DOI:** 10.1186/1878-5085-5-S1-A85

**Published:** 2014-02-11

**Authors:** T Kezeli, T Rukhadze, N Gongadze, G Sukoyan, N Dolidze, M Chipashvili, M Mirsiashvili

**Affiliations:** 1 Department of pharmacology, Faculty of Medicine, State University, Tbilisi, Georgia; 2 Department of pharmacology, State Medical University, Tbilisi, Georgia

## Introduction

Calcitonin gene-related peptide (CGRP) is one of the most potent vasodilator, which dilates multiple vascular beds, but the coronary circulation is particularly sensitive. The purpose of this study was to evaluate the influence of CGRP antagonist on: 1. Acute mortality; 2. Ventricular arrhythmias and; 3. Prostaglandin E_2_ (PGE_2_) production by nitrate induced tolerance rats with myocardial infarction (MI).

## Methods

Experiments were carried out in anaesthetized male Wistar rats (BW)-250-300g. MI was induced by ligation of left coronary artery (CAL) resulting in a large anterolateral MI. 3 groups of rats were treated: group 1 received only nitroglycerin (NTG, 50mg/kg.d, subcutaneous injections b.i.d) 3 days prior to MI; group 2 received NTG in the same dose, route and frequency of administration + CGRP antagonist (CGRP_8-37_) - 10 mcg/kg 2 times daily, by similar period of administration; group 3 rats served as normal control (C). ECG tracing in lead II was obtained permanently before induction of MI and up to 60 min postinfarction. At 60 min postinfarction 1 ml of blood was drawn from the femoral artery for analysis of PGE_2_ by radio immunoassay.

## Results

CAL after 60 min was accompanied by high incidence of venticular arrhytmias (VA) associated with a significant mortality in group 1 (72%) and especially in group 2 (88%) rats as compared with C group of animals (60%) correlated to profound decrease in PGE_2_ content more markedly in group 2 (2.2±0.4ng/ml) vs. group 1 rats (3.6±0.2ng/ml, p<0.05) and vs. C group (4.8±0,3ng.ml, p<0.001) respectively.

## Conclusion

It is suggested that CGRP may be important mechanism of nitrate tolerance and diminution availability of CGRP (as potent vasodilator) may lead to exacerbation of acute MI.

